# Impact of menaquinone-4 supplementation on coronary artery calcification and arterial stiffness: an open label single arm study

**DOI:** 10.1186/s12937-016-0175-8

**Published:** 2016-05-12

**Authors:** Yuji Ikari, Sho Torii, Atsushi Shioi, Toshio Okano

**Affiliations:** 1 Department of Cardiovascular Medicine, Tokai University School of Medicine, 143 Shimokasuya, Isehara, 259-1193 Japan; 2 Department of Cardiovascular Medicine, Osaka City University Graduate School of Medicine, 1-5-7 Asahimachi, Abenoku, Osaka, 545-0051 Japan; 3 Department of Hygienic Sciences, Kobe Pharmaceutical University, 4-9-1 Motoyamakitamachi, Higashinadaku, Kobe, 658-0003 Japan

**Keywords:** Vitamin K, Coronary artery calcification, Pulse wave velocity

## Abstract

**Background:**

Dietary intake of vitamin K has been reported to reduce coronary artery calcification (CAC) and cardiovascular events. However, it is unknown whether supplemental menaquinone (MK)-4 can reduce CAC or arterial stiffness. To study the effect of MK-4 supplementation on CAC and brachial ankle pulse wave velocity (baPWV).

**Methods:**

This study is a single arm design to take 45 mg/day MK-4 daily as a therapeutic drug for 1 year. Primary endpoint was CAC score determined using 64-slice multislice CT (Siemens), and the secondary endpoint was baPWV measured before and 1 year after MK-4 therapy.

**Results:**

A total of 26 patients were enrolled. The average age was 69 ± 8 years and 65 % were female. Plasma levels of phylloquinone (PK), MK-7, and MK4 were 1.94 ± 1.38 ng/ml, 14.2 ± 11.9 ng/ml and 0.4 ± 2.0 ng/ml, respectively, suggesting that MK-7 was the dominant vitamin K in the studied population. Baseline CAC and baPWV were 513 ± 773 and 1834 ± 289 cm/s, respectively. At 1 year following MK-4 supplementation, the values were 588 ± 872 (+14 %) and 1821 ± 378 cm/s (−0.7 %), respectively. In patients with high PIVKA-2, −18 % annual reduction of baPWV was observed.

**Conclusion:**

Despite high dose MK-4 supplementation, CAC increased +14 % annually, but baPWV did not change (−0.7 %). The benefits of MK-4 supplementation were only observed in patients with vitamin K insufficiencies correlated with high PIVKA-2 baseline levels, reducing baPWV but not CAC.

**Trial registration:**

This study was registered as UMIN 000002760

## Introduction

Coronary artery calcification (CAC) forms in the pathogenesis of atherosclerosis [1] and is associated with a higher risk of cardiovascular events [2, 3]. Annual changes of CAC-scores are considered to be relevant with severity of atherosclerosis [1, 2]. The vitamin K dependent Matrix Gla protein (MGP) plays a role as an inhibitor of soft tissue calcification [1, 4–6]. Patients with therapeutic vitamin K antagonist tended to have more valvular, vascular and coronary calcification [7, 8]. Observational studies in humans showed an inverse relationship between menaquinone (MK) intake and CAC in healthy elderly [9, 10]. Phylloquinone (PK) supplementation was shown to retard the progression of CAC and had a beneficial effect on vascular stiffness in healthy adults with coronary artery calcification after 3 years of follow-up [11–13]. A randomized, double-blind, placebo-controlled trial to investigate the effect of menaquinone-7 (MK-7) supplementation on MGP species showed a dose-dependent decrease of dephospho-uncarboxylated MGP (dp-ucMGP) concentrations [14]. Furthermore, MK-7 improves arterial stiffness and elastic properties of the carotid artery [15]. These data may suggest that vitamin K administration may have beneficial effects on the vasculature. Supplementation studies using MK-4 has been few. This pilot study analyzed the impact of MK-4 supplementation on CAC and arterial stiffness.

## Methods

### Patient selection and study protocol

Patients with at least one coronary risk factor (coronary risk factors were defined as hypertension, diabetes mellitus, hypercholesterolemia, smoking, and family history of coronary artery disease) were enrolled. Exclusion criteria were patients with implantation of coronary stent or pacemaker, or inability to obtain correct coronary artery calcification (CAC) score or brachial ankle pulse wave velocity (baPWV) data. Written informed consent was obtained from each participant. The Institutional Review Board approved the study and all patients gave written informed consent. Medical histories, including prior myocardial infarction, prior percutaneous coronary intervention, prior coronary artery bypass graft surgery, prior heart failure, prior stroke, and hemodialysis, were obtained for each patient. The correlation between coronary artery calcification score, aortic stiffness and each factor was studied.

### Menqauinone-4 treatment

A tablet containing 15 mg of MK-4 (Eizai, Tokyo) was prescribed three times a day. This drug is approved for osteoporosis and is commercially available in Japan. If any side effects of the MK-4 treatment occurred, it was to be reported to the study center. In the case of newly onset atrial fibrillation, MK-4 could be stopped if vitamin K antagonist was indicated to prevent stroke.

### Cardiac multi-slice computed tomography data acquisition and analysis

In all patients, a prospective non-enhanced coronary calcium scan was performed with a 64-slice MSCT scanner (Siemens, Munich, Germany). For quantitative assessment of coronary artery calcification, the Agatston score [16] was calculated, using a 3 mm CT slice thickness and a detection threshold of 130 Hounsfield units (HU) involving ≧ 1 mm^2^ area/lesion (3 pixels). Total CAC score was determined by summing individual lesion scores from each of four anatomic sites (left main trunk, left anterior descending artery, left circumflex artery, and right coronary artery) [17]. The measurement was performed using syngo calcium scoring software supplied by Siemens. The inter-observer and intra-observer errors were reported as coefficient of variation of 2.1 and 1.3 [18]. CT was performed before starting MK-4 and 1 year after MK-4 treatment.

### Measurement

Plasma was obtained from the patients in the morning after overnight fasting and stored at – 30 °C. Vitamin K (PK, MK-4, and MK-7) was determined by the high-performance liquid chromatography-tandem mass spectrometry (LC-APCIMS/MS) method [19]. Total circulating uncarboxylated matrix gla protein (t-ucMGP) measurements were done by Dr. Vermeer’s group. Intact parathyroid hormone, osteocalcin (OC), ucOC, NTX and bone type alkaline phosphatase (BAP) and high sensitive C-reactive protein were measured by SRL Inc (Tokyo, Japan). Bone density was measured at lumbar vertebra using DSC-900FX (Hitachi-Aloka Medical, Tokyo). The ankle brachial index (ABI) and baPWV were measured using BP-203 RPE (Omron-Colin, Kyoto).

### Endpoints

The primary endpoint of this study was CAC score difference between baseline and 1 year after MK-4 treatment. The secondary endpoint was baPWV difference. Other plasma data and clinical data were obtained at baseline.

### Statistics

We present continuous variables as mean ± standard deviation in normal distribution or median and interquartile range. Categorical variables were presented as absolute numbers and percentages. Statistical analysis was performed with SAS version 9.2, (SAS Institute, Inc., Cary NC). This study was registered as UMIN 000002760.

## Results

A total of 26 patients were enrolled. Baseline characteristics are shown in Table 1. The average age was 69 ± 8 years and 65 % were female. Diabetes was 15 % and the ankle brachial index was 1.11 ± 0.39. Baseline baPWV was 1834 ± 289 and the CAC score was 513 ± 773 (median 264 [48–484]).

**Table 1:** Patient background

Number	26
Female gender	65 %
Age	69 ± 8
Height	157 ± 9
Weight	57 ± 11
Body mass index	22.8 ± 3.2
Current smoker	27 %
Diabetes mellitus	15 %
Hypertension	73 %
Dyslipidemia	81 %
History of myocardial infarction	15 %
Prior coronary artery bypass surgery	4 %
History of stroke	4 %
Ankle brachial index	1.11 ± 0.39
ba-pWV (cm/s)	1834 ± 289
Bone matrix	1.053 ± 0.251
% Bone matrix	117 ± 20 %
Coronary artery calcium score	658 ± 1049
Medications	
Aspirin	42 %
Statin	58 %
ACEI or ARB	54 %
Calcium antagonist	58 %
beta blocker	19 %
Insulin	8 %

*ba-pWV* brachial ankle pulse wave velocity

*ACEI* angiotensin converting enzyme inhibitor

*ARB* angiotensin receptor blocker

Baseline blood test data are shown in Table 2. Uncarboxylated osteocalcin (ucOC) was 3.7 ± 2.5 ng/ml and PIVKA2 was 19 ± 7 mAU/mL. Plasma levels of PK, MK-7, and MK4 were 1.94 ± 1.38 ng/ml, 14.2 ± 11.9 ng/ml and 0.4 ± 2.0 ng/ml, respectively. This suggests that MK-7 was the dominant vitamin K in the studied population.

**Table 2:** Baseline data

Hemoglobin (g/dL)	13.8 ± 1.2
Albumin (g/dL)	4.2 ± 0.3
Triglyceride (mg/dL)	136 ± 82
alkaline phosphatase (IU/L)	223 ± 59
Blood urea nitrogen (mg/dL)	15 ± 3
Creatinine (mg/dL)	0.74 ± 0.24
ucOC (ng/ml)	3.7 ± 2.5
OC (ng/ml)	7.5 ± 2.7
ucOC/OC ratio	0.46 ± 0.18
PIVKA2 (mAU/mL)	19 ± 7
intact parathyroid horomone (pg/mL)	38.9 ± 22.0
Bone specific alkaline phosphatase (μg/l)	13.8 ± 6.5
NTX (nmol BCE/L)	16.9 ± 5.1
high sensitive C-reactive protein (mg/L)	929 ± 1132
Osteoprotegerin (ng/mL)	91.7 ± 30.8
oxidized low density lipoprotein (μg/dL)	104.9 ± 11.9
t-ucMGP (nmol/L)	2907 ± 1333
PK (ng/mL)	1.94 ± 1.38
MK-7 (ng/mL)	14.2 ± 11.9
MK-4 (ng/mL)	0.4 ± 2.0

*ucOC* uncarboxylated osteocalcin

*OC* osteocalcin

*PIVKA2* protein induced by vitamin K absence or antagonist- 2

*NTX* collagen type 1 cross-linked N-telopeptide

*t-ucMGP* total circulating uncarboxylated matrix gla protein

*PK* philloquinone, *MK* menaquinone

**Fig. 1:**
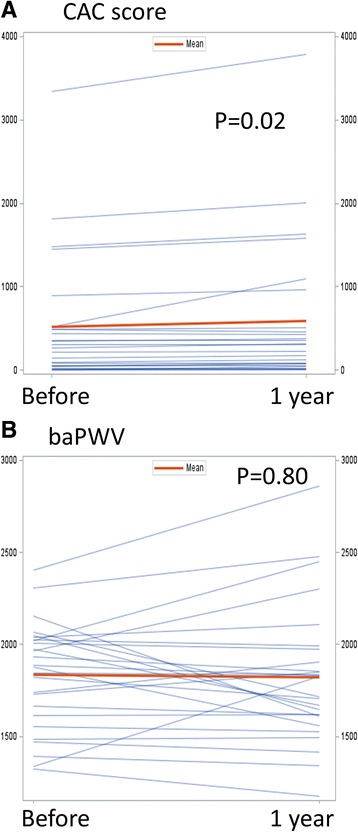
**a** Paired profiles of coronary artery calcium scores before and after MK-4 supplementation. **b** Paired profiles of pulse wave velocities before and after MK-4 supplementation

**Fig. 2:**
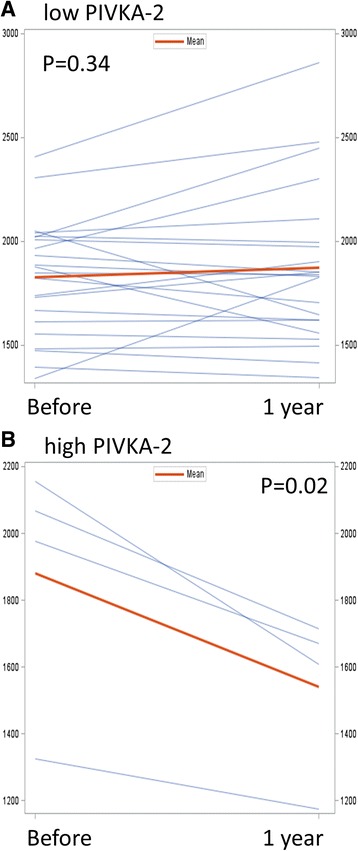
**a** Paired profiles of pulse wave velocities before and MK-4 supplementation in patients with PIVKA-2 < 23. **b** Those in patients with PIVKA-2 > 23

**Fig. 3:**
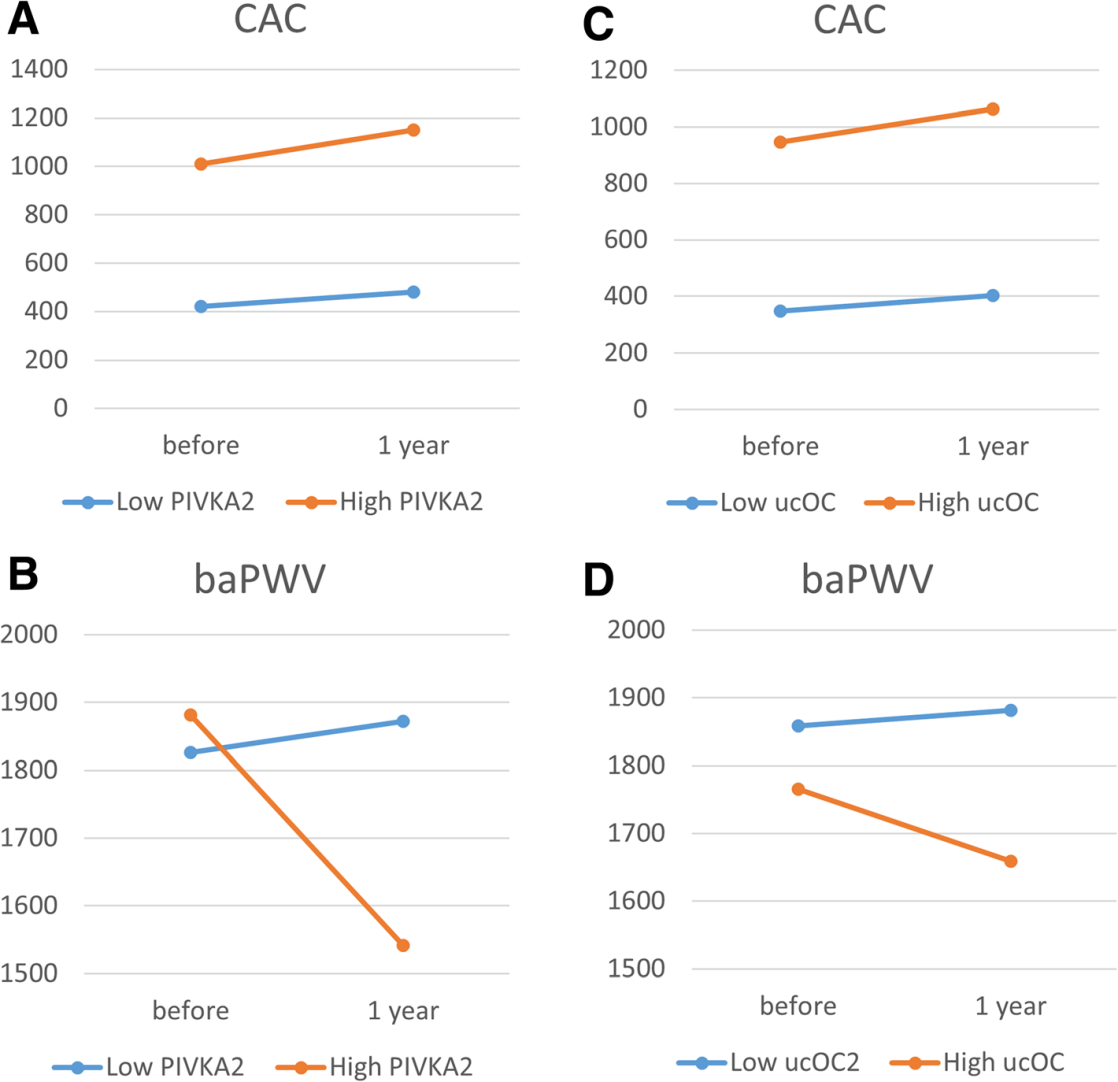
Average values of CAC and baPWV before and after 1 year MK-4 supplementation. **a** CAC and (**b**) baPWV stratified by PIVKA-2 level. **c** CAC and (**d**) baPWV stratified by ucOC level

CAC and baPWV data before and after MK-4 treatment are shown in Table 3. CAC significantly increased despite the MK-4 treatment and baPWV did not change (Fig. 1). The patients were divided into categories of baseline vitamin K insufficiency levels based on PIVKA-2 or ucOC indicators. Regardless of the baseline level of PIVKA-2 or ucOC, CAC similarly increased in each group. To the contrary, baPWV was reduced significantly by MK-4 supplementation in patients with a high PIVKA-2 baseline (Fig. 2). However, the reduction was not observed in patients with low PIVKA-2. A similar pattern was observed with baseline ucOC levels, but it was not statistically significant (Fig. 3).

**Table 3:** Change after 1 year MK-4 supplementation

**CAC**		N	Pre	1 year	Difference	%Difference	Paired *P* value	Baseline *P* value
Total		26	513 ± 773	588 ± 872	72 ± 143	+14 %	0.018	
PIVKA2	≦23	22	421 ± 543	481 ± 610	60 ± 132	+14 %	0.052	0.64
PIVKA2	>23	4	1010 ± 1558	1151 ± 1761	141 ± 204	+14 %	0.26	
ucOC	<4.5	19	348 ± 448	402 ± 515	55 ± 139	+16 %	0.11	0.38
ucOC	≧4.5	7	947 ± 1232	1064 ± 1387	117 ± 157	+12 %	0.09	
**PWV**		N	Pre	1 year	Difference		Paired *P* value	Baseline *P* value
Total		26	1834 ± 289	1821 ± 378	−12.7 ± 263	−0.7 %	0.811	
PIVKA2	≦23	22	1826 ± 280	1872 ± 379	47 ± 226	+2.6 %	0.34	0.79
PIVKA2	>23	4	1882 ± 379	1542 ± 248	−340 ± 164	−18 %	0.026	
ucOC	<4.5	19	1859 ± 293	1882 ± 410	22 ± 261	+1.2 %	0.71	0.26
ucOC	≧4.5	7	1766 ± 288	1659 ± 220	−107 ± 240	−6.1 %	0.28	

Despite 1 year MK-4 supplementation, CAC increased +14 % annually. High PIVKA2 and high ucOC indicate vitamin K insufficiency at baseline. Those with high PIVKA2 had significant reduction of PWV

## Discussion

Despite high dose MK-4 supplementation, CAC increased +14 % annually, but baPWV did not change (−0.7 %). CAC similarly increased annually irrespective of baseline vitamin K insufficiency. The MK-4 supplementation improved baPWV only in patients with vitamin K insufficiency.

An annual increase of CAC was reported as 17 % in the meta-analysis [20]. The mean annual CAC-progression reported in the literature ranges from 24 to 51 % and has a large inter-individual variation depending on many factors such as the baseline CAC-score, medical history, medication-use, body-mass index, scanner type and manufacturer [21]. This study showed 14 % CAC increase annually, however it is unknown whether MK-4 supplementation retarded progression because this study lacks the control group. At the very least, MK-4 did not stop CAC progression.

MK-7 supplementation significantly decreased dephospho-uncarboxylated MGP dose-dependently [14]. MK-7 supplements may help postmenopausal women to prevent bone loss [22]. Low-dose menaquinone-4 improves gamma-carboxylation of osteocalcin in young males [23]. Animal studies showed that high-dose MK-7 supplementation inhibits the development of cardiovascular calcification in rats [24]. Shea et al. reported that there was no difference in CAC progression between PK supplementation group and control group; the mean (±SEM) changes in Agatston scores were 27 ± 6 and 37 ± 7, respectively. A 270-day course of low-dose vitamin K2 (90 μg/day, MK-7) administration in patients with CKD stages 3–5 may reduce the progression of atherosclerosis, but does not significantly affect the progression of calcification [25]. On the other hand, this study is as high as high dose (45 mg/day, MK-4) The effective dose to protect atherosclerosis or calcifications is unknown.

In terms of arterial stiffness, long-term use of MK-7 supplements improves arterial stiffness in healthy postmenopausal women, especially in women having a high arterial stiffness [15]. The results of this study were similar to prior reports as have little preventive effects on CAC. On the other hand, effects on arterial stiffness were also observed in this study.

There are several limitations of this study. This study was a small number, single center and a single arm study. It is difficult to judge the effect of MK-4 because of the lack of a control group.

In conclusion, despite MK-4 supplementation, CAC progressed 14 % annually. The arterial stiffness was not changed overall, but reduction was observed only in patients with baseline vitamin K insufficiency.

## Abbreviations

baPWV: brachial ankle pulse wave velocity
CAC: coronary artery calcification
dp-ucMGP: dephospho-uncarboxylated matrix gla protein
MK: menqauinone
PIVKA-2: protein induced by vitamin K absence or antagonist- 2
PK: phylloquinone
ucOC: uncarboxylated osteocalcin
